# Density of tobacco retail outlets around school in Lao People's Democratic Republic 2024: a geospatial analysis

**DOI:** 10.3389/fepid.2026.1817143

**Published:** 2026-06-01

**Authors:** Shweta Kulkarni, Thanh Cong Bui, Phonepadith Xangsayarath, Khatthanaphone Phangdouangsy, Chanthavy Soulaphy, Khanittha Sengdara, Sydney Martinez, Amanda Janitz, Summer G. Frank-Pearce, Laura A. Beebe

**Affiliations:** 1 Department of Biostatistics and Epidemiology, Hudson College of Public Health, University of Oklahoma Health Campus (OUHC), Oklahoma City, OK, United States; 2 The Matilda Centre for Research in Mental Health and Substance Use, The University of Sydney, Sydney, OK, Australia; 3 Department of Family and Preventive Medicine, College of Medicine, University of Oklahoma Health Campus (OUHC), Oklahoma City, OK, United States; 4 TSET Health Promotion Research Center, Stephenson Cancer Center, OUHSC, Oklahoma City, OK, United States; 5 National Center for Laboratory and Epidemiology, Ministry of Health of Lao People’s Democratic Republic (Lao PDR), Vientiane, Lao PDR; 6 Secretariat of the National Tobacco Control Committee, Ministry of Health of Lao PDR, Vientiane, Lao PDR

**Keywords:** geographic information system, geospatial analysis, Lao People's Democratic Republic, tobacco prevention, tobacco retail density

## Abstract

**Introduction:**

Exposure to tobacco advertising at tobacco retail outlets (TROs) is associated with smoking initiation among youth. There is limited geospatial evidence on the density of TROs in Lao People's Democratic Republic (PDR), a country with high prevalence of tobacco smoking. This study examined the density and proximity of TROs around schools in two urban districts and one rural district of Vientiane Capital, Lao PDR, using geographic information systems.

**Methods:**

We audited 233 TROs around 27 schools between January 19 and February 18, 2024. TROs were mapped within 250 m and 500 m buffers in two urban districts (Chanthabuly and Sissatanak), and 500 m and 1,000 m buffers in one rural district (Naxaithong). Buffer analysis and network analysis estimated TRO density and median walking distances between TROs and schools. We used the Kruskal–Wallis test to determine if TRO density varied significantly in urban districts within two buffers and the chi-square test to examine differences in TRO characteristics based on proximity to schools.

**Results:**

TRO density was defined as the number of TROs mapped within a 250 m radius of urban schools and those mapped within 1,000 m radius of rural schools. TRO density was higher within the buffer of 250–500 m in Chanthabuly (median = 12), followed by Sissatanak (median = 3) (*p* = 0.01). Comparing the two urban districts, the median distance between TROs and schools within the buffer of 250–500 m was significantly less in Sissatanak compared to Chanthabuly (*p* = 0.04). The shortest distance between an urban school and any TRO (i) without age verification signage was 21.58 m, (ii) with outside cigarette advertisements was 9.95 m, and (iii) selling “single” cigarettes was 311.36 m. In the rural district, the TRO density was higher within 500–1,000 m (median = 2) compared to within 500 m (median = 0.5).

**Conclusion:**

Within the context of the Lao PDR, our study provides the first geospatial evidence of tobacco retail outlet density in both urban and rural districts of Vientiane Capital, revealing a substantially higher concentration of outlets in urban districts.We also quantified walking distances between schools and outlets violating tobacco control measures, including lack of age verification signage and outdoor cigarette advertising. These findings suggest that stricter regulation of tobacco retail outlets could strengthen tobacco control policy implementation in Lao PDR.

## Introduction

Tobacco retail density can influence smoking behaviors. A high tobacco retail density is associated with high availability of cigarettes and ease of purchase (1), promoting a cultural norm that smoking is acceptable (2). Evidence suggests that a higher tobacco retail density in a neighborhood is correlated with higher purchase attempts, smoking frequency, and lifetime smoking among youth (3). A higher tobacco retail density is also associated with lower perceived risks of smoking among youth leading to increased smoking prevalence (4). Areas with higher tobacco retail density often experience weaker enforcement of age restrictions, which makes it easier for minors to purchase tobacco products (5). In addition, exposure to point-of-sale (POS) tobacco advertising and the absence of age verifications at tobacco retails are associated with smoking initiation (6). Examining retail density, particularly around schools, can provide valuable insight into areas with high retail density, and help identify locations with tobacco control violations may be occurring. Moreover, recent geospatial studies (7–9) employing simulation-based evaluations of tobacco retail regulations have enabled the assessment of multiple tobacco control policies, underscoring the critical role of geospatial approaches in advancing understanding of, and informing regulations within, the tobacco retail environment.

Tobacco smoking remains a major cause of death and disability in the Association of Southeast Asian Nations (ASEAN) (10), a region consisting of ten countries (11). In Lao People's Democratic Republic (PDR), adult male smoking is very high at 51%, second only to Indonesia (63%), while the adult female smoking rate is the highest (7.1%) followed by the Philippines (5.8%) (12). Evidence from Southeast Asia, particularly the ASEAN region, shows that tobacco retail density is higher in urban areas compared to rural areas. Comparing ASEAN countries is appropriate because they share a heavy, interconnected tobacco-related disease burden and face similar challenges, including strong tobacco-industry lobbying and economic dependence on tobacco farming (13). For example, evidence in Indonesia suggests a higher density of outdoor tobacco advertisements in urban settings (14). Evidence also indicates a high tobacco retail density near schools, including finding of 40% higher tobacco retail density close to educational facilities in Indonesia (15). Two studies in Bali, reporting that schools had at least one TRO within 250 m (m) (3) (16), suggesting widespread availability of tobacco retail outlets near schools. Similar evidence has been documented in Thailand (17). However, for the remaining ASEAN region, including Lao PDR, there is limited evidence on the spatial distribution of tobacco retail outlets near schools and on potential violations in tobacco control measures around schools.

The World Health Organization (WHO) Framework Convention on Tobacco Control (FCTC) is the first international treaty negotiated under the auspices of WHO, developed in response to the globalization of the tobacco epidemic (18), with Lao PDR adopting it in September 2006 (18). Despite this commitment, there is limited evidence on tobacco retail environment in Lao PDR. One study examined the prevalence of cigarette sale, and found differences in cigarette prices in urban and rural areas (19), but no research has examined the tobacco retail density or proximity to schools in Lao PDR (20). The Global Youth Tobacco Survey, in Lao PDR, in 2016 found that 35.2% of students aged 13–15 years were exposed to POS advertising or promotions (21). Moreover, among the children who tried to buy cigarettes, more than half were not prevented from buying them because of their age (21). These findings highlight the need to examine the tobacco retail density near schools in Lao PDR particularly, to generate evidence on potential tobacco control violations near schools.

We conducted a cross-sectional examination of tobacco retail stores around schools in Lao PDR to examine the tobacco retail density around schools in two urban and one rural district of Vientiane capital city of Lao PDR. We also examined the proximity of tobacco retail outlets to schools using geographic information systems (GIS). Our study provides the first geospatial evidence of tobacco retail outlet density in urban and rural Lao PDR with evidence on potential violations in tobacco control measures near schools.

## Methods

### Study design

We canvased the selected study area in two urban and one rural district of Vientiane Capital in Lao PDR. We conducted observational surveys to collect data on tobacco retail outlets (TRO) characteristics and geospatial data on TROs and schools between January 19 and February 18, 2024.

We used a two-stage cluster sampling approach with a non-random selection of clusters to define our catchment area. In first stage, we identified two urban districts, namely, Chanthabuly and Sissatanak, and one rural district of Naxaithong in the Vientiane Capital of Lao PDR based on population density of districts (22), and based on logistical constraints of time and resources. In the second stage, within each of the identified district, we identified TROs by defining catchment areas around schools (secondary schools, high schools, and colleges) (23). These TROs served as the secondary sampling units from which data was collected.

There was no list of licensed TROs in Lao PDR. As per the Lao Ministry of Education 2023–2024, there were 17 schools in Chanthabuly, 21 schools in Sissatanak, and 16 schools in Naxaithong district (a total of 54 schools). Due to the lack of precise street-level addresses for schools (24), we were able to map only a limited number of schools (27 in total). The smallest administrative unit in the available geospatial data was at the district level (25), rather than the street-level, as observed in other GIS-based studies in Lao PDR (26). Our study sample consisted of 27 schools in three districts.

We selected schools based on following inclusion criteria,

1. **Mapping of locations**: Schools listed on the Ministry of Education website whose locations could be mapped using publicly available street addresses on Google Maps. Since only province- and district-level school data was available (24), this led to inclusion of 7 schools in Chanthabuly, 5 schools in Sissatanak, and 6 schools in Naxaithong (a total of 18 schools in 3 districts) (marked with * in Supplementary Table S1) (24, 27).
2. **Logistical constraints**: Only those schools were mapped that were within the logistical constraints related to time and resource availability.
3. **Private or international schools** in urban districts were included that were not listed on the Ministry of Education website but with publicly available street addresses, resulting in the inclusion of 4 schools in Chanthabuly and 5 in Sissatanak.
4. **Nature of schools**: Secondary and high schools with student age ranges of 11–18 and 18–24 years, respectively (25), reflecting typical ages for smoking initiation (21, 26, 27).

Some challenges that led to the exclusion of certain schools included the unavailability of precise street addresses on Google Maps, particularly for schools in the rural district of Naxaithong, limited time and resources for extended field visits, and inconsistencies in school names on Google Maps that did not allow the mapping of certain schools (22). Our study selected schools based on logistical feasibility and access constraints, and this purposive selection may limit the representativeness of our study findings.

### Sampling method

A 500 m radius was defined and canvased around each school in the two selected urban districts. In the rural district, a radius of 1,000 m defined the catchment area. We calculated TRO within 250 m and 500 m of schools in the urban district and within 500 m and 1,000 m of schools in the rural district. We chose a larger radius in the rural district since the schools and TROs in rural district are located at considerable distances from each other (28). The catchment areas were defined based on similar distances around schools in other studies (3, 29). Different buffer distances were applied in urban and rural settings to reflect systematic differences in built-environment characteristics, particularly the greater spatial separation between schools and tobacco retail outlets in rural districts compared with urban areas. Because tobacco retail outlets captured within different buffer distances are inherently and systematically different in scale and context, direct quantitative comparisons across non-comparable buffers were avoided to minimise potential bias.

We compiled our survey based on two validated tools, the Standardized Tobacco Assessment for Retail Settings (STARS), used in the US to inform POS tobacco control policies (30), and the Environmental Profile of a Community's Health (EPOCH) survey (31) used to assess tobacco policy implementation in low- and middle-income countries that have ratified the WHO FCTC (31). Our final survey included four sections: Product, Placement, Price, and Targeting Youth. The “Product” assessed the presence of PHWs on cigarette packs and the number of cigarettes per pack. The “Promotion” section assessed the presence of cigarette advertising outside the store and branded materials inside stores. The “Price” section recorded the brand and price of cigarettes sold, whether there was sale of single cigarettes. The “Targeting Youth” examined the sale of tobacco products within 12 inches of youth-friendly items such as candies, gum, cold drinks, etc., and the display of tobacco products within 3 feet of the floor.

The TROs audited and mapped were visible from the streets or alleys. TROs were defined as any stores that sold consumer goods, which included kiosks, restaurants and food vendors, street vendors, mini markets, and supermarkets. We also included TROs that were visible from the street (16). No formal permission was requested from the TRO owners or staff before data collection. Data collectors entered TROs as regular customers and data were collected through direct observation without interfering with the usual operations of TROs.

We used a Garmin GPSMAP 67i portable GPS device to collect the GPS coordinates of the schools and TROs. The GPS device was satellite-based and had sufficient battery life to support data collection. All the GPS coordinates were recorded within 0.5 to 1 m from the main entrances of the schools and TROs. Prior to the field audit, the school locations were mapped using their addresses on Google Maps to define catchment areas for identifying TROs. During the audit, geolocations of the schools were also recorded using a GPS device to ensure spatial accuracy. While there was a difference of only a few degrees between the geo-coordinates obtained from Google Maps and those recorded by the GPS device, we utilized the more accurate GPS coordinates for our analysis.

The primary author (SK) collected data with the help of local research staff. The team walked and used private transportation to cover the streets in the catchment area. Geocoordinates of schools and TROs were recorded with a precision of ≤10 m of the respective locations. We made observations at the TROs between 9 am–6 pm to match the opening hours of most TROs. Study data were collected and managed using REDCap electronic data capture tools hosted at the University of Oklahoma Health Center (32, 33).The data collectors received training on using the GPS device and the tobacco audit procedure. The team conducted a mock audit of the TROs to ensure a complete understanding of the audit protocol. We performed quality control by revisiting TROs (5%–10% of our sample).

### Analysis

For mapping, we obtained district-level geospatial data from the Humanitarian Data Exchange (HDX), Lao PDR, subnational administrative boundaries, 2024 (25). Our geospatial analytical approach aligns with the recent tobacco retail environment studies utilizing ArcGIS Pro, HDX administrative boundaries, and school-centred buffer analysis approach (7) We projected the data using the WGS 1984 World Equidistant Cylindrical projection. The GPS device collected data in Degrees, Minutes, Seconds (DMS) system. The GPS data for schools and TROs were cleaned and converted from DMS to decimal degrees (DD) (34). The data cleaning and management were performed in Microsoft Excel, and analyses were performed using ArcGIS Pro software 3.3.0 (35).

The TRO density was defined as the median number of TROs within a certain buffer distance of schools (250 m and 500 m for urban districts and 500 m and 1,000 m for the rural district), aligning with recent evidence (7)We reported median density with the interquartile range since our data were not normally distributed (15).

We defined the TRO proximity to school as (i) the median of Euclidean Distance (in m) and interquartile range between a TRO and the school within two defined school buffers (250 m and 500 m) in urban districts, (ii) the median distance (in m) and interquartile range between the two closest TROs within two defined school buffers (250 m and 500 m) in urban districts.

We categorized the proximity distances into ≤100, 100–250, and >250 m, similar to a previous study (15) and based on regulations banning TROs within 100 m of schools in Indonesia (3). We calculated the shortest distance between schools and TROs within the buffers of 250 m and 500 m. This provided proximity between schools and TROs, considering real-world travel conditions (17).

We conducted a school-centred buffer analysis by creating two sets of circular areas around schools: one set with radii of 250 m, another set at 251–500 m for urban districts, and another set with radii of 0–500 m and 501–1,000 m for the rural district. We determined the number of TROs between the 250 m and 500 m buffers for urban districts. Since TROs located in overlapping buffer areas might be counted multiple times, we used a summarization method such that we counted TROs more than once when located in overlapping buffers. Finally, we categorized the TROs based on their location relative to the buffers and the characteristics of both the TROs and schools. Overlapping buffers were intentionally permitted in our geospatial analysis. A single TRO could fall within the buffer zones of multiple schools, and in such cases, TROs were counted multiple times. This approach was adapted because the study analyses and sampling aimed to assess the school-specific exposure to tobacco retail environment. Tobacco retail density was calculated by counting TROs multiple times, consistent with our school-centred framework. Our approach is consistent with other school-centred geospatial tobacco retail studies (8, 36).

We conducted a network analysis using the Origin Destination Cost Matrix in ArcGIS Pro v. 11.4 (ESRI, Redlands, CA) to calculate the walking distance in meters between schools and tobacco retail outlets (TROs) and neighboring TROs. The analysis ranked the shortest travel distances from each school to each tobacco shop using street network data. We used street networks since we used the Open Street Map on the Garmin device. We focused on walking as the mode of travel, reflecting how data collectors physically walked to the sites during the audit. No restrictions or time-based calculations were applied, as the goal was to identify the shortest walking distances and highlight the accessibility of tobacco products to students (37).

To compare the two urban districts, we performed the Kruskal Wallis non-parametric test to (i) test if the TRO densities (median number of TROs per school) within a school buffer vary significantly (tested separately for 250 m and 250–500 m), (ii) test if the median distance between the TROs and schools within each buffer varies significantly between two urban districts, and (iii) if the distance between the two closest TROs within a school buffer varies significantly between two urban districts (tested separately for within 250 m and 250–500 m).

Using the urban district data, we performed a chi-square test to examine TRO characteristics in the urban districts, such as age verification signage, presence of PHWs on packs, and presence of cigarette advertisements and promotions differed significantly based on the proximity to the school (≤250, and >250 ≤ 500 m). If more than 20% of cells have expected frequencies less than 5 in the contingency tables, we used Fisher's Exact Test. We used SAS vs. 9.4 (Cary, NC) for statistical analysis.

## Results

We mapped and observed 213 TROs around 27 schools in the 3 districts of Chanthabuly (105 TROs around 11 schools), Sissatanak (89 TROs around 10 schools), and Naxaithong (19 TROs around 6 schools). We performed quality control by revisiting TROs (5%–10% of our sample).

### TRO density around schools

Comparing the two urban districts, the density of TROs was highest at 250–500 m around schools in Chanthabuly (median = 12), compared to schools in Sissatanak (median = 3). This difference was statistically significant (*p* = 0.01). Within the 250 m buffer, the median number of TROs was 7 in Chanthabuly and 2.5 in Sissatanak, but this difference was not statistically significant. (*p* = 0.14). Although there were 213 unique TROs, many were in overlapping buffers and counted more than once for some analyses. In the rural district of Naxaithong, TRO density was low within both buffers around schools (Table 1).

**Table 1:** Density of tobacco retail outlets (TROs) around schools in urban, rural districts of Lao people's democratic republic, 2024.

Buffer distances around schools	Number of TROs per school within a buffer around schools^a^ Median (Interquartile range)				
	Buly (Urban) *n* = 105	Sissatanak (Urban) *N* = 89	Kruskal Wallis test *P* value**	Buffer distances around schools	Naxaithong (Rural) *n* = 19
Within 250 m of the school	7 (3–13)	2.5 (1–6)	0.13	Within 500 m of school	0.5 (0–3)
>250–500 m from school	12 (4–24)	3 (2–6)	**0**.**01**	>500–1,000 m from school	2 (1–4)
Number of schools covered	11	10		Number of schools covered	6
Total number of unique TROs audited within 500 m of schools during audit	105	89		Number of unique TROs within 1,000 m of schools during audit	19
The ratio of the number of TROs to the number of schools	10:1	9:1		The ratio of the number of schools to the number of TROs observed	3:1
Total number of TROs within 500 m counted within multiple school buffers (some duplicated)	229	170		Total number of TROs within 1,000 m counted within multiple school buffers	20

a Number of TROs calculated using Buffer Analysis such that TROs are counted more than once when located in overlapping buffers.

** *P* value for Kruskal Wallis test to examine if median number of TROs within each school buffer (250 m & 250–500 m) varies significantly between the two urban districts.

Bold values denote statistically significant results (*p* < 0.05).

### Proximity of TROs to schools across districts

We did not draw comparisons in the TRO density and proximity to schools between the urban and the rural district due to differences in the sampling methodology of the schools and TROs across the urban and rural districts (250 m and 500 m for urban districts and 500 m and 1,000 m for the rural district).

Comparing the two urban districts, the median distance between TROs and schools within the 250–500 m buffer was significantly shorter in Sissatanak (366.06 m) compared to Chanthabuly (406.51 m) (*p* = 0.01). In the rural district of Naxaithong, the median distance between the TROs and schools within the 250–500 m buffer was 676.03 m.

Comparing the distance between the two closest TROs in school buffers across the urban districts, Sissatanak had more densely populated TROs, with the median distance between the two closest TROs being 20.17 m compared to Chanthabuly where the median distance was 50.01 m, and the difference was statistically significant (*p* = 0.04). However, the same difference was not significant when comparing the two urban districts within the buffer of 250–500 m. In the rural district of Naxaithong, the distance between the two closest TROs was 734.69 m within the buffer of 500–1,000 m (Table 2).

**Table 2:** Proximity of tobacco retail outlets (TROs) to schools in urban and rural districts, Lao people's democratic republic, 2024.

Proximity measures of TROs	Buffer distances around the schools	Chanthabuly (Urban) Median (Interquartile range)	Sissatanak (Urban) Median (Interquartile range)	Kruskal Wallis test^a^ *P* value***	Buffer distances around the schools	Naxaithong (Rural) Median (Interquartile range)
Distance between TROs and schools within the school buffer (in meters) (Median, Range) ^b^	Within 250m of the school	89.44 (27.09–211.17)	156.34 (108.15, 207.10)	0.165	Within 500 m of school	148.44 (2.89, 74.42)
	>250–500 m from school	406.51 (341.69–444.77)	366.06 (351.05–389.82)	**0.01**	>500–1,000 m from school	676.03 (569.79–782.28)
Distance between the two closest TROs within the school buffer (in meters) (Median, Range) ^c^	Within 250 m of the school	50.01 (2.81–140.89)	20.17 (0–169.35)	**0.04**	Within 500 m of school	37.39 (0–11.47)
	>250–500 m from school	415.55 (370.67–465.84)	402.91 (331.95–460.38)	0.06	>500–1,000 m from school	734.69 (661.86–883.82)

a Kruskal Wallis test used to compare the median distances between schools and TROs in urban districts only.

b Distances of TROs from schools were calculated using Network Analysis such that a unique distance (in meters) is calculated between TROs and schools within a district.

c Distances between the two TROs calculated using Network Analysis such that a unique distance (in meters) is calculated between the two TROs.

*** *P* value for Kruskal Wallis test to examine if the median distance between (“TRO and school” or “two closest TROs”) varies significantly between two urban districts across each school buffer.

N represents counts.

Bold values denote statistically significant results (*p* < 0.05).

### TRO characteristics based on proximity to schools

In our network analysis was used to examine the proximity of TROs to schools based on TRO characteristics, we did not observe statistically significant differences between TROs located within ≤250 m of schools and those situated >250–500 m in urban districts (all *p* > 0.05).

Despite the absence of statistically significant associations, the analyses suggested a greater presence of certain TRO characteristics in the >250–500 m buffer compared with the ≤250 m buffer. Specifically, TROs located >250–500 m from urban schools tended to more frequently lack age-verification signage, sell cigarette packs without pictorial health warnings, and display outdoor cigarette advertisements relative to those situated within ≤250 m of schools (Table 3).

**Table 3:** Tobacco retail outlet (TRO) characteristics (n, %) within the two urban districts by distance to the closest school in Lao people's democratic republic, 2024^a^.

TRO characteristics	Number of unique TROs in urban districts (*N* = 194)		Chi-squared test P** value	Walking distance between the audited TROs to the nearest school (in meters), calculated using Network analysis
	≤250 m (*N* = 56) N, %	>250 ≤ 500 m (*N* = 138) *N*, %		Median (Minimum, Maximum)
No age verification signage	22 (39.30)	61 (44.20)	0.75	350.72 (21.58, 490.32)
Cigarette packs without pictorial health warnings for sale	7 (12.55)	23 (16.66)	0.71***	345.38 (63.59, 497.99)
Cigarette advertisements outside the store	10 (17.85)	32 (23.18)	0.57***	335.22 (9.956, 497.84)
Branding materials inside the store	19 (33.92)	30 (21.73)	0.20	414.17 (170.40, 482.48)
“Single” cigarettes for sale	0	2 (1.44)	ND	311.36 (311.36, 311.36)
Alcoholic beverages for sale along with tobacco products	27 (48.21)	73 (52.89)	0.48	353.85 (9.95, 497.99)
Tobacco products within 12 inches of candy, gum, etc.	30 (53.57)	80 (57.97)	0.46	351.90 (9.95, 497.80)
Tobacco product advertisements within 3 ft of the floor	31 (51.35)	81 (58.69)	0.58	351.05 (9.60, 497.99)

a Number of TROs are calculated based on their unique distances from schools, with those distances calculated using Network Analysis;.

** *P value* for Chi-Squared test to examine if the proportion of TROs differ significantly based on buffer distances from school (tested for each of the TRO characteristic separately);.

*** *P* value for Fisher Exact test (when more than 20% of cells have expected frequencies less than 5 in the contingency tables) examining if the proportion of TROs differ significantly based on buffer distances from school; N and % represent count and percentage respectively; ND represents “Not Defined”.

Although these differences were not statistically significant, there was a pattern indicating that a greater proportion of TROs located 250–500 m from urban schools sold tobacco products within 12 inches of youth-appealing items such as candy and gum compared with TROs located within ≤250 m. A similar pattern was observed for tobacco product advertisements positioned within 3 ft of the floor, with slightly higher proportions among TROs located 250–500 m from schools relative to those within ≤250 m (Table 3). These findings should be interpreted with caution, as the lack of statistically significant differences may be reflected due to potential absence of differences in tobacco retail characteristics across different buffers. Accordingly, these results are presented as descriptive and future studies with larger and more representative samples may have sufficient power to formally assess whether statistically significant differences in tobacco retail outlet characteristics exist across buffer distances from schools.

The shortest walking distance calculated using network analysis between a school and (i) a TRO without age verification signage was 21.58 m, (ii) a TRO selling cigarette packs without PHWs was 63.59 m, and (iii) a TRO with outdoor cigarette advertisements, it was 9.95 m. We observed 2 TROs selling “loose” cigarettes in the rural district and the shortest walking distance to any school was 311.36 m (Table 3**)**.

**Figure 1:**
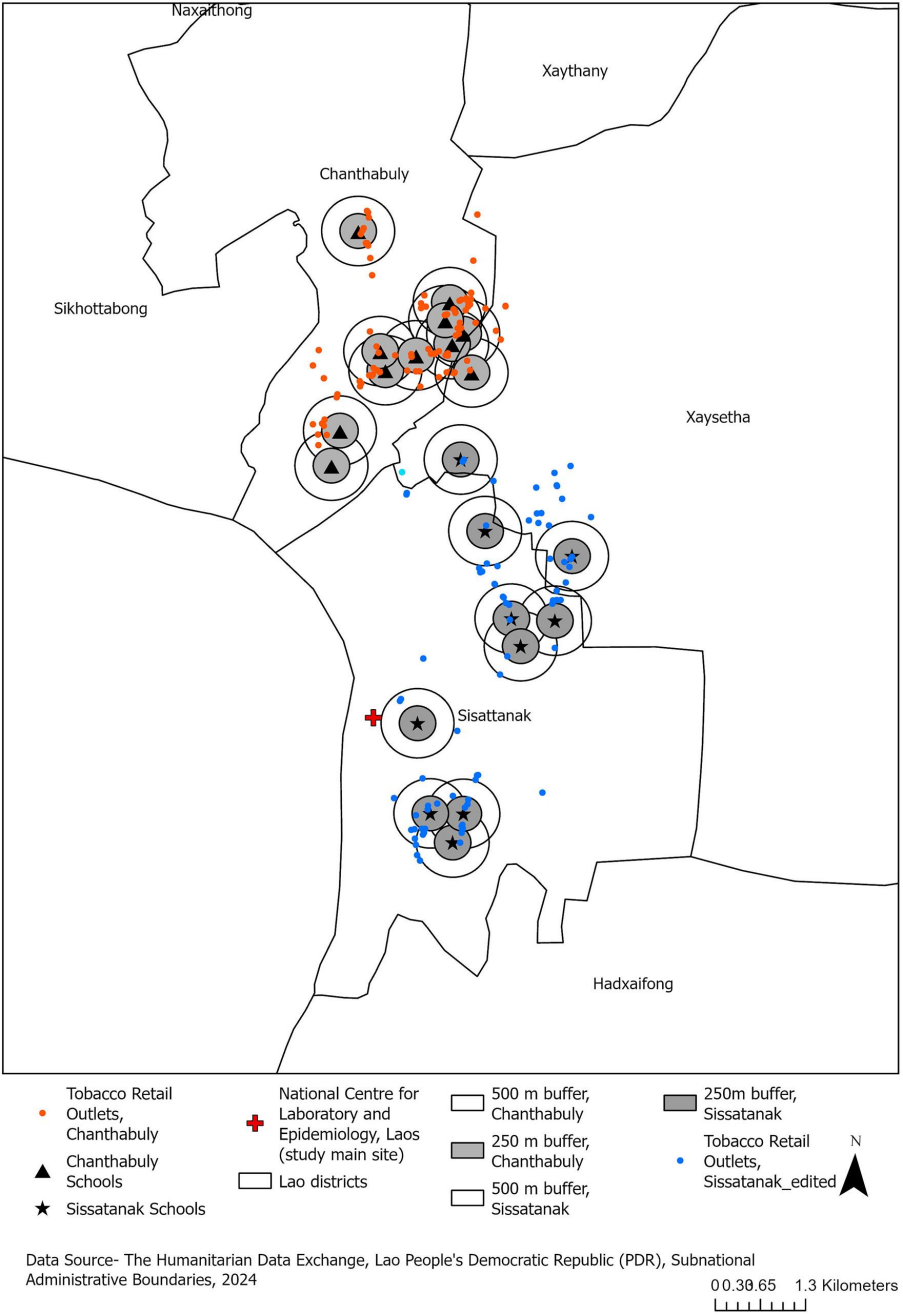
Results of buffer analysis showing tobacco retail outlets within the 250 m and 500 m buffers around schools in the two urban districts of chanthabuly and sissatanak, Lao People’s democratic republic, 2024. Related analyses and visualisations have been published previously (Kulkarni S. et. al, Front. Public Health, 2026).

**Figure 2:**
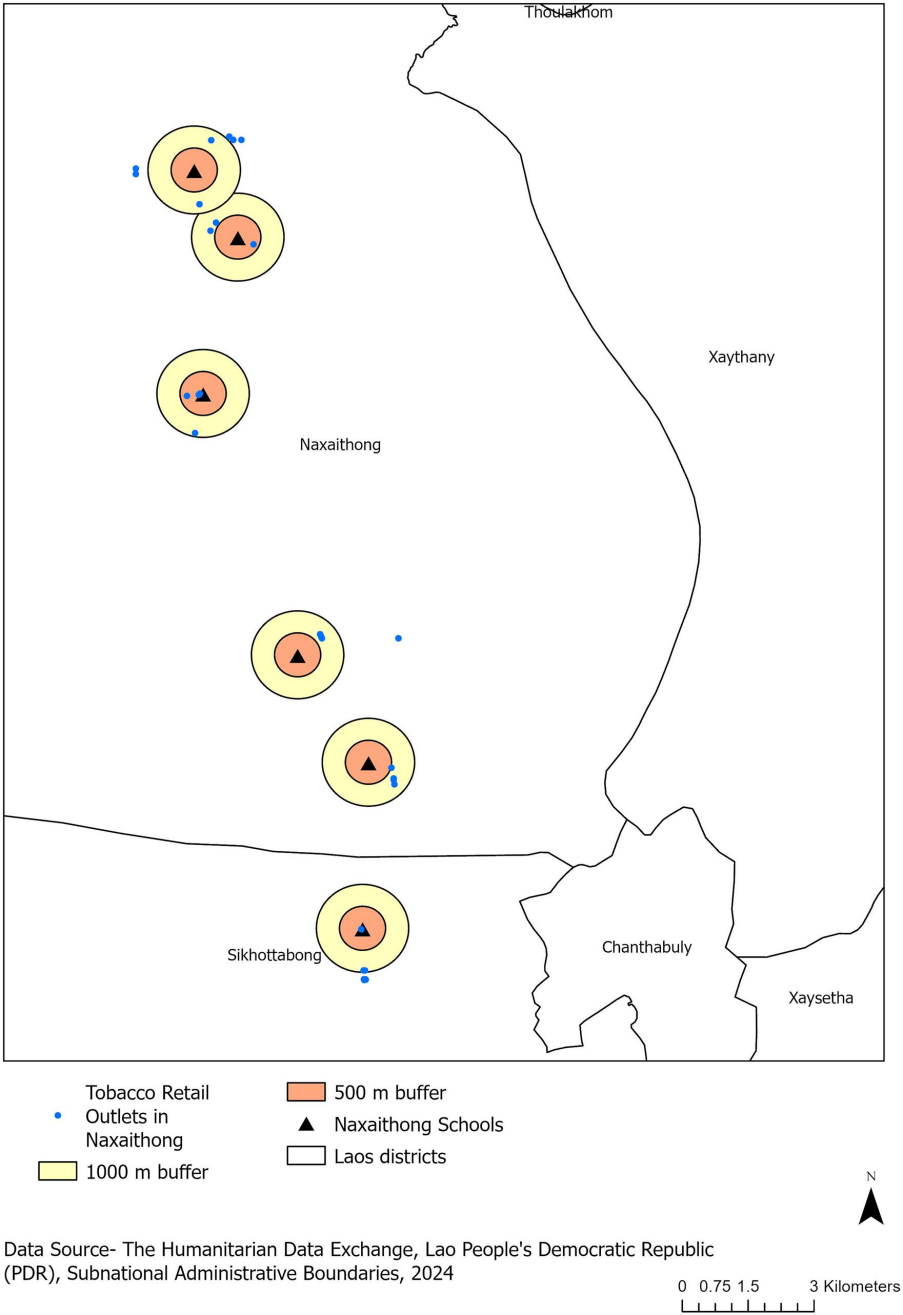
Results of buffer analysis showing tobacco retail outlets within the 500 m and 1,000 m buffers around schools in the rural district of naxaithong, Lao People's democratic republic, 2024. Related analyses and visualisations have been published previously (Kulkarni S. et. al, Front. Public Health, 2026).

Figure 1 shows the location of TROs within the 250 m and 500 m buffers around schools in the two districts of Chanthabuly and Sissatanak. Figure 2 shows the location of TROs within 500 m and 1,000 m of schools in the rural district of Naxaithong in Lao PDR. The fact that two schools in the urban district of Sissatanak and one school in the rural district of Naxaithong appear to lie beyond the borders of study districts can be attributed to differences in the resolution and precision of the geographic datasets used.

## Discussion

This study is the first in the Lao PDR to examine tobacco retail outlet (TRO) density and their proximity to schools using geospatial analysis.We found a high density of TROs near schools in urban districts. We found 10 TROs located within 500 m of one school in Chanthabuly and 9 within the same distance of a school in Sissatanak, resulting in TRO-to-school ratios of 10:1 and 9:1, respectively. These findings align with a study from Thailand, which reported a ratio of 8:1 within 500 m of schools (14). Additionally, we found, nearly a quarter (23.5%) of TROs located within 100 m of schools displayed outdoor tobacco advertisements. Also, we found TROs lacking age verification signage were found as close as 350.72 m from schools, while those with outdoor advertisements were located as near as 335.22 m. Our findings are consistent with findings from Indonesia, where TROs with advertisements were observed within 300 m of primary and high schools (38). This cross-sectional evidence from our study provides evidence of ongoing tobacco advertising and lack of age verifications at TROs near schools suggesting that stricter regulations may be beneficial to prevent tobacco exposure near schools in Lao PDR.

In Lao PDR, gaps in the tobacco advertising, promotion, and sponsorship (TAPS) ban, particularly the allowance of point-of-sale advertising and certain promotions, remain a policy concern (39). Consistent with this, our study observed point-of-sale advertising in retail outlets. Despite legal prohibitions on sales to individuals under 18 and bans on single cigarette sales (39), we identified a lack of age verification practices near at some TROs, suggestive of inadequate tobacco control measures near schools.

Our study found that two rural TROs sold “single” cigarettes, found the median distance to a school was 311.36 m. This finding suggests the potential for easy availability of cigarettes to school students. Our findings are supported by another study in Indonesia which found TROs selling “single” cigarettes to young people at a distance >500 m from school (3). Similarly, we found that the nearest TRO to a school in the rural district of Naxaithong was 2.89 m and in the urban district of Sissatanak was 9.96 m. Our findings are comparable to evidence in Indonesia that found TROs within 1.7 m of school) (16) and TROs were within 2.9 m of school (3). Our study findings provide cross-sectional evidence suggesting that stronger enforcements may be beneficial to limit tobacco product availability near schools.

School zoning policies banning tobacco sales near schools have been shown to reduce youth access to cigarettes (40). Evidence from Indonesia (3), and Thailand (17) suggest that banning tobacco retail within 500 m of schools could reduce youth exposure to tobacco advertisements.

Article 21 of the Amended Tobacco Control Law requires licenses for sale and distribution of tobacco products (38). While our study could not verify tobacco retailer's licensing status, it identified violations of tobacco control policies. Future studies should evaluate the implementation of tobacco retail licensing in Lao PDR.

Our study is observational in nature and provides empirical, context-specific evidence describing the spatial distribution and proximity of tobacco retail outlets to schools in Lao PDR. Some recent simulation-based geospatial studies in Egypt (7) and studies from the US (8, 9) extend observational evidence by modelling the potential effects of specific tobacco control policies, such as retailer buffer zones, licensing caps, operating-hour restrictions, and age-restricted sales locations. These studies represent methodological strengths by integrating policy simulation, scenario testing, and geospatial analyses to estimate the impact of regulatory interventions. Our study approach is complementary to these studies as our findings map tobacco retail concentration and youth proximity as a policy-relevant problem. Simulation-based studies could further demonstrate how targeted regulatory strategies could substantially reduce tobacco availability. Our study builds on established methodologies used in low- and middle-income countries (7) while extending prior work through the incorporation of network-based analyses and field-verified GPS locations of tobacco retailers, enabling a more context-specific assessment of the retail environment around schools.

### Future directions

Future research in Lao PDR and comparable settings could build on these methodological advances by applying simulation-based geospatial models to evaluate country-specific tobacco retail policies and support evidence-informed tobacco control planning. Further work could assess tobacco retail outlet density around schools in diverse geographic settings, examine underlying drivers of urban–rural disparities in retail density, and explore associations between retail exposure and tobacco use among youth attending nearby schools. Although Lao PDR has adopted tobacco retail licensing in line with WHO FCTC Article 15 (39), strengthening the retail licensing could improve monitoring while also applying penalties for retailers selling non-compliant tobacco products. Future geospatial studies, in the context of Lao PDR, could use more granular spatial units (e.g., provincial rather than district-level data) enabling more precise estimation of tobacco retail density and exposure patterns.

### Limitations

This study had several limitations-

1. There might be systematic differences between the TROs included in our study and the TROs in the rest of the districts of Lao PDR, leading to selection bias in our findings, limiting the generalizability of study findings beyond the Vientiane city. Additionally, some TROs within 500 m of schools may have been missed, potentially leading to an underestimation of TRO density. Future studies could include more diverse sample of districts thereby more precisely examining the tobacco retail density across urban and rural districts of Lao PDR.
2. The schools in the study were included based on logistical feasibility and access constraints. This purposive selection of the schools may limit the representativeness of the study findings beyond the selected schools and districts of Lao PDR. Future studies could include a broader and diverse sampling of schools.
3. The inclusion of additional private and international schools in the urban districts could have led to inclusion of additional urban TROs that wouldn't be included otherwise, potentially overestimating the tobacco retail density near those schools. Our study couldn't measure the tobacco retail density specific to school type owing to lower sample size, future studies could measure the tobacco retail density and proximity to schools based on different school types.
4. There might be a bias introduced by the potential differences in the data recording procedures among different data collectors. We attempted to minimize this bias by training the data collectors and performing a quality check of the recorded data.
5. We did not collect data on youth tobacco purchasing patterns, preventing analysis of the relationship between TRO density and youth tobacco use.
6. **Geospatial limitations**: The Origin Destination Cost Matrix Analysis used in our study, has been used in a wide variety of global settings such as a matrix for infrastructure and public transport analysis outside the US including in Indonesia (41) and in China (42), justifying its use. However, the open street map data obtained using the GPS device may not be as detailed in rural or remote parts of Lao PDR as in developed countries such as the US. While Garmin GPS devices provide precise location points, if the open street map network is outdated or incomplete it may affect the accuracy of the network analysis. There is a possibility that the administrative boundaries obtained from the data source, the Humanitarian Data Exchange (HDX) (version 2.2.3): https://data.humdata.org/dataset/cod-ab-lao, may not be detailed enough, which may affect our analysis results. Also, the data resolution from HDX might not align perfectly with the more granular GPS data. Such discrepancies may have introduced errors in precise geospatial alignment while projecting the GPS device data and HDX data on Arc GIS Pro. These discrepancies and data misalignment could explain why some of the TROs were projected in neighboring districts, even though they are located in the same district (as seen in Figure 1). This could lead to an underestimation of the TRO density around that specific school.

The geospatial analyses in our study were limited to comparisons within similar buffer distances in urban districts. The relatively limited sample size constrained our ability to conduct robust comparisons across all buffer distances, which should be considered when interpreting the findings. Future studies with larger and more representative samples could examine whether meaningful differences exist in tobacco retail outlet density and characteristics across varying distances from schools.

Given the absence of statistically significant differences across buffer distances in the present study, future studies with larger and more representative samples could apply similar geospatial methods to determine whether meaningful differences in tobacco retail outlet characteristics exist across distances from schools.

In our study, two of the schools are projected in neighboring urban district of Xaysetha instead of the urban district of Sissatanak (as seen in Figure 1), however both the schools are officially listed under the Sissatanak district as per the Ministry of Education, Laos (43). Therefore, this discrepancy does not affect the implications of our study, which are to estimate the TRO density and proximity around schools in Sissatanak. Similarly, another school was projected in the suburban district of Sikhottabong instead of the suburban district of Naxaithong (as seen in Figure 2), but the school is officially listed under Naxaithong as per the Lao Ministry of Education (43) suggesting a milder impact on study implication. We used the “district” level geospatial data for our analysis, which may not be the best unit to show differences in the TRO densities.

## Conclusion

Despite these limitations, within the context of the Lao PDR, our study provides the first observational geospatial evidence of tobacco retail outlet density and proximity to schools in a country with a high smoking prevalence. Our study demonstrated a high tobacco retail density in urban districts. We also quantified walking distances between schools and tobacco retail outlets violating tobacco control measures, including lack of age verification signage and outdoor cigarette advertising. These findings suggest that stricter regulation of tobacco retail outlets could strengthen tobacco control policy implementation in Lao PDR.

## Data Availability

The original contributions presented in the study are included in the article/Supplementary Material, further inquiries can be directed to the corresponding author/s.
